# Luminescence nanothermometry using a trivalent lanthanide co-doped perovskite

**DOI:** 10.1039/d2ra05935e

**Published:** 2023-01-18

**Authors:** Prashansha Singh, Neha Jain, Shraddha Shukla, Anish Kumar Tiwari, Kaushal Kumar, Jai Singh, Avinash C. Pandey

**Affiliations:** a Nanotechnology Application Centre, University of Allahabad Prayagraj 211002 UP India prashansha26singh@gmail.com +91 9452105068; b Department of Physics, Dr Harisingh Gour Central University Sagar 470003 MP India; c Department of Physics, IIT (ISM) Dhanbad 826004 Jharkhand India; d Department of Pure & Applied Physics, Guru Ghasidas Vishwavidyalaya (A Central University) Bilaspur 495009 India; e Inter University Accelerator Centre Aruna Asaf Ali Marg New Delhi 110067 India

## Abstract

This study investigates in detail the laser-mediated upconversion emission and temperature-sensing capability of (Ca_0.99−*a*_Yb_0.01_Er_*a*_)TiO_3_. Samples were prepared at different concentrations to observe the effect of erbium on upconversion while increasing its concentration and keeping all the other parameters constant. Doping is a widespread technological process which involves incorporating an element called a dopant in a lower ratio to the host lattice to derive hybrid materials with desired properties. The (Ca_0.99−*a*_Yb_0.01_Er_*a*_)TiO_3_ perovskite nanoparticles were synthesized *via* a sol–gel technique. The frequency upconversion was performed using a 980 nm laser diode excitation source. X-ray diffractometry (XRD) confirmed that the synthesized samples are crystalline in nature and have an orthorhombic structure. The temperature-sensing ability was examined using the fluorescence intensity ratio (FIR) algorithm of two emission bands (^2^H_11/2_ → ^4^I_15/2_ and ^4^S_3/2_ → ^4^I_15/2_) of the Er^3+^ ion. Temperature-dependent upconversion luminescence is observed over a broad temperature range of 298–623 K. The maximum sensor sensitivity obtained is 6.71 × 10^−3^ K^−1^ at 110°.

## Abstract 2

This study investigates in detail the laser-mediated upconversion emission and temperature-sensing capability of (Ca_0.99−*a*_Yb_0.01_Er_*a*_)TiO_3_.

## Graphical abstract

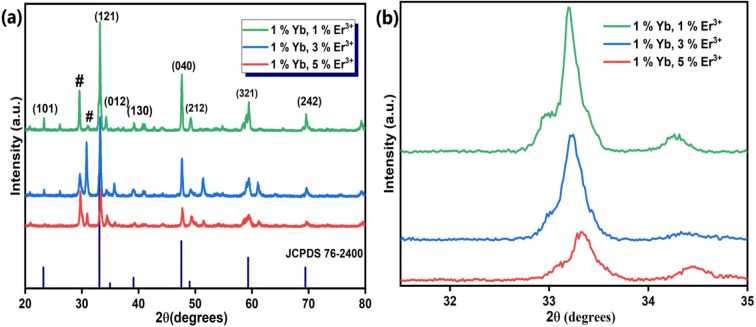

## Introduction

Rare earth doped/codoped upconversion luminescent materials are a unique class of nanophosphors which when excited by a suitable energy, emit a photon in the visible range. To obtain efficient upconversion (UC) emission it is essential to choose an excellent host material. CaTiO_3_ has wide band gap (3.8 eV–4.3 eV),^1^ which allows emission from embedded luminescent centers mediated by effective excitation. The perovskite compounds of ABO_3_-type structure have been considered promising inorganic functional materials due to their stable crystal structure.^2–4^ To date many researchers have investigated perovskite-based temperature sensors but with lower temperature-sensing performance.^5^ The interest in CaTiO_3_ perovskite is growing with the passage of time because of its unique properties, such as: high chemical durability, high thermal stability, being cheaper than sulfides or nitrides, and being less reactive than fluorides. In this present study, CaTiO_3_ is considered as a host owing to the remarkable properties above and its high refractive index.^6^ Moreover, it has a phonon frequency low enough to achieve good upconversion intensity. UC luminescence is in general observed in lanthanide ions owing to their ability for f–f transition along with numerous metastable energy states.^7^ Optical UC is an anti-Stokes process which upconverts two or more low-energy photons into one high-energy photon.^8^ Lanthanide ions doped into a suitable host lattice show multiple advantages, such as longer luminescence lifetimes, lower toxicity and high photochemical stability.^9–13^

Er^3+^ is one such member of the lanthanide family, which shows sharp emission. This acts as a green emission activator ion which has well-known intermediate energy levels which are capable of being populated using near infrared excitation.^14,15^ Yb^3+^ is a very good sensitizing agent owing to its suitably broad absorption cross-section over the near-infrared region, and it is also able to transport its energy to the activator ion by an excited-state absorption route. Considering the above advantages of lanthanides as luminescent centers and perovskites as host materials, it is an obvious idea to work on their combination: *i.e.* this combination has the ability to offer upconversion photoluminescence for wide applications in several fields: for example, display devices, drug delivery, cancer therapy, biological imaging solar light conversion, optical thermometry, and security applications.^16–21^

Precise evaluation of temperature is required in several industrial fields, to obtain a desired progression, and it is being developed under most favorable conditions. Temperature sensing is one such facilitatory non-contact temperature calculation method with fast response, high measurement accuracy and high spatial resolution.^22,23^ There are several methods to measure temperature sensing ability,^24^ and the most frequently used are the fluorescence lifetime (FL) and fluorescence intensity ratio (FIR) of thermally coupled and/or non-thermally coupled electronic transitions of rare earth ions.^25–31^ The FIR method is an effective and well-recognized method for reliable and accurate measurements because it is functional over a broad range of temperature.^32^ In this technique, the essential requirement is intensity variation in UC luminescence caused by coupled electronic transitions to a low-energy state from two closely spaced high-energy states. The population rate becomes low with higher energy levels due to the huge energy gap between the thermally coupled levels, and thereby emission intensity from these levels is reduced. The thermally coupled levels are in general found in trivalent lanthanide ions *e.g.* Sm^3+^, Eu^3+^, Tm^3+^, Er^3+^, Ho^3+^ and Nd^3+^.^14,33–35^ In particular, FL-based optical thermometry is not dependent on outer intrusions or changes in excitation density and is thus suitable for use in severe environmental conditions, such as chemical reaction temperature monitoring, microwave induction heating or radio frequency and plasmas.^36,37^

Photoluminescence (PL) emission intensity starts decreasing beyond a certain proportion of rare earth, and this trend is known as luminescence quenching. Luminescence quenching occurs because of an increasing rate of non-radiative decay from the excited state.^38^ Furthermore, talking about temperature-dependent UC, when heating a phosphor, PL emission intensity increases up to a definite temperature; but if the phosphor is heated further at higher temperatures, a decline in the intensity can be observed.^39^ This may be due to non-radiative relaxation (increasing rate of phonon vibration) of excitons at raised temperatures, reducing the intensity.

## Materials

CaCl_2_ (HiMedia, 97.7%), Yb_2_O_3_ (Merck, 99.998%), Er_2_O_3_ (Merck, 99.9%), titanium(iv) bis (acetylacetonate) diisopropoxide (Merck), HNO_3_ (70%), citric acid (99.5%), ethanol (99.9%), and ammonia (70%) were procured commercially. We took CaCl_2_ (0.98 M) = 2.1754 g, Er_2_O_3_ (0.01 M) = 0.076 g, Yb_2_O_3_ (0.01 M) = 0.078 g and 3 mL of titanium(iv) bis(acetylacetonate) diisopropoxide solution for CaTiO_3_: 1 at% Er^3+^, 1 at% Yb^3+^. For other samples we calculated the amount of 3% Er^3+^ and 5% Er^3+^ using the formula (Ca_0.99−*a*_Yb_0.01_Er_*a*_)TiO_3_.^40^ The chemicals were used as received without further refinement.

## Experimental methods

To synthesize (Ca_0.99−*a*_Yb_0.01_Er_*a*_)TiO_3_ with constant Yb^3+^ and varied concentrations of Er^3+^ (at molar ratios of 1, 3 and 5%), the well-recognized sol–gel technique was used. To start with, appropriate amounts of CaCl_2_, Er_2_O_3_, and Yb_2_O_3_ were added to a beaker containing 20 mL of deionized water, and a few drops of nitric acid were added. The obtained precursor solution was then stirred vigorously at a rate of 400 rpm along with constant heating at 100 °C, until a transparent solution was obtained. In the subsequent step, 3 mL of titanium solution was taken in another beaker containing ethanol solution and 0.4 M of citric acid and stirred, for only a few minutes. Then, both solutions were added together, which then turned into a precipitate. The precipitate was heated for 1 h, and washed with alcohol for removal of any impurities present. We obtained a xerogel after drying the precipitate at 100 °C for 12 h under constant heating. A dried black powder was obtained after the gel was baked at 400 °C for 2 h. Finally annealing was performed at 900 °C, giving a white crystalline powder as the end product.

The synthesized samples were examined for frequency UC-emission using a 980 nm diode laser (continuous mode, power tuneable) as the excitation source and a dispersive monochromator (model: iHR320, Horiba Jobin Yuon) equipped with a photomultiplier tube (model no. R928P, Hamamatsu, Japan) was used as a detector. In order to investigate the microstructure, a JEOL JEM-F200 TEM (transmission electron microscope), equipped with SAED (selected area electron diffraction) was used. HRTEM (high-resolution TEM) was used to record the atomic planes inside the crystal lattice. The X-ray diffraction patterns were measured on a PANalytical Advanced X-ray diffractometer using a Cu Kα (1.5406 Å) radiation source over the angular range 20° ≤ 2*θ* ≤ 80°.

## Results and discussion

### X-ray diffraction

**Fig. 1:**
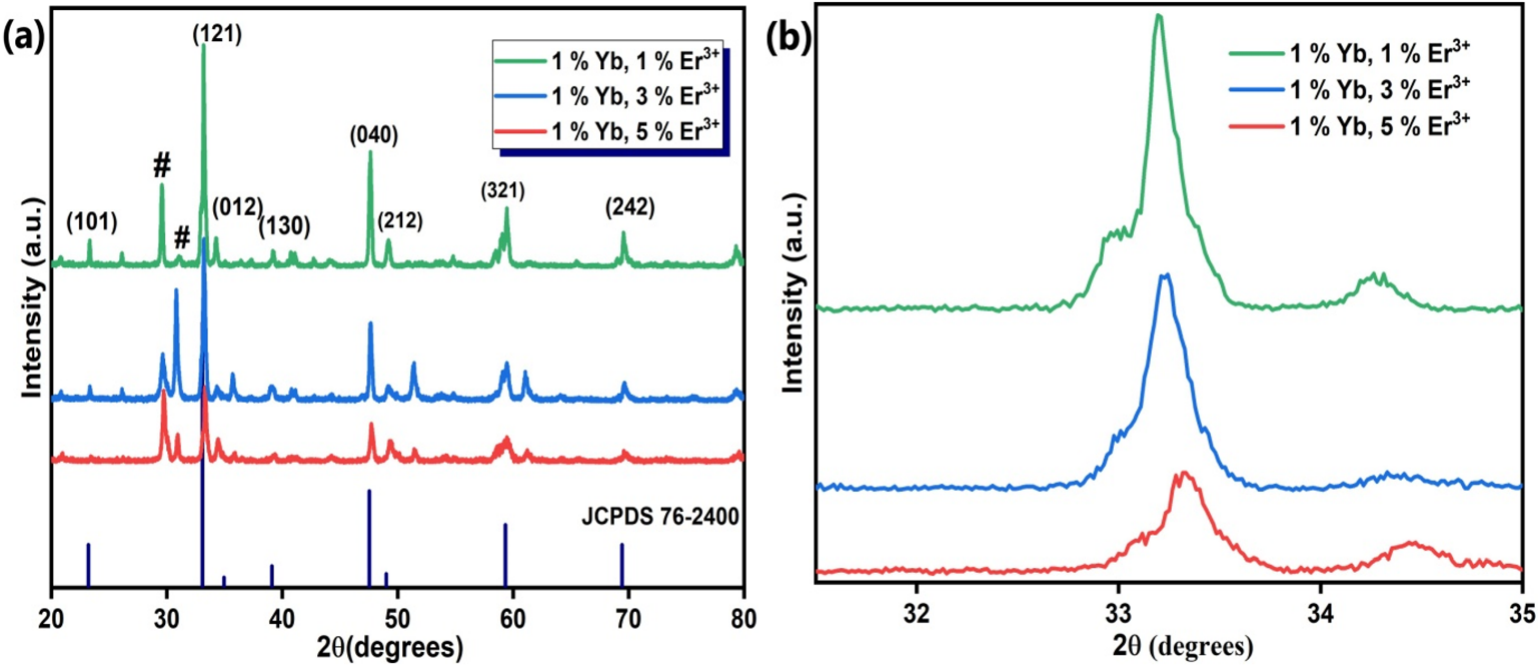
(a) X-ray powder diffraction pattern of as-synthesized (Ca_0.99−*a*_Yb_0.01_Er_*a*_)TiO_3_ (where *a* = 0.01, 0.03, 0.05). (Vertical drop lines symbolize standard JCPDS card number 76-2400.) (b) shift in (121) peak with Er^3+^ incorporation.

The XRD patterns are presented in Fig. 1(a). The diffraction peaks (101), (121), (012), (130), (040), (212), (321) and (242) can be well indexed to pure CaTiO_3_, matching well with standard JCPDS card number 76-2400, and exhibit an orthorhombic crystal structure with space group *Pcmn*(62). The peak at 2*θ* = 33.21° exhibits maximum intensity. However, there are some extra peaks at 29.58° and 31.05° with weak intensity marked by #. These peaks are a result of the formation CaTiO_3_ matching the orthorhombic phase of CaTiO_3_. The outcomes of the XRD data are very consistent with the TEM/SEM results. We evaluated the crystallite size of the samples using the Scherrer formula, given as:1
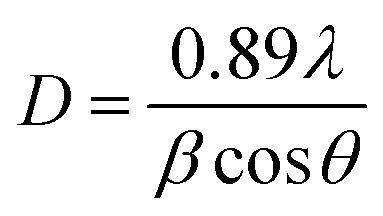
where *D* stands for average crystallite size, *λ* represents the wavelength of the X-rays (1.5406 Å for the Cu Kα source), *β* stands for FWHM (full width at half maxima) in radians and *θ* is the diffraction angle. The average crystallite sizes calculated using the above formula are 33.14 nm, 23.60 nm, and 20.82 nm for the CaTiO_3_: 1 at% Er^3+^, 1 at% Yb^3+^, CaTiO_3_: 3 at% Er^3+^, 1 at% Yb^3+^ andCaTiO_3_: 5 at% Er^3+^, 1 at% Yb^3+^ samples, respectively. The trend in crystallite size shows that with increasing concentration of erbium the crystallite size decreases. This change can be attributed to the change in lattice parameters caused by substitution of Ca^2+^ ions (ionic radius = 2.31 Å) with smaller radius ions Er^3+^ (ionic radius ≈ 0.89 Å) and Yb^3+^ (ionic radius ≈ 0.86 Å).

We used the Williamson–Hall relation^41^ to determine the strain present in the lattice, which is given below:2
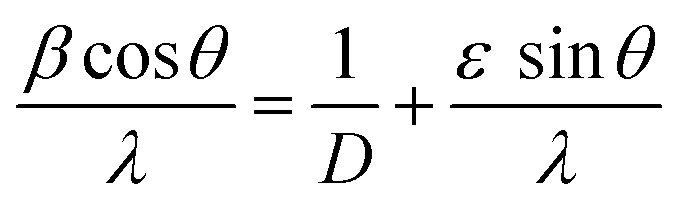
where the terms are same as in the Scherrer formula above. The strain appears due to distortion of the lattice in the crystal. The respective strains present for the as-synthesized samples CaTiO_3_: 1 at% Er^3+^, 1 at% Yb^3+^, CaTiO_3_: 3 at% Er^3+^, 1 at% Yb^3+^ and CaTiO_3_: 5 at% Er^3+^, 1 at% Yb^3+^ are 3.49 × 10^−3^, 4.48 × 10^−3^ and 5.27 × 10^−3^. From Fig. 1(b) it can be observed that there is a slight shift in peak position towards higher 2*θ* angle while the peak intensity decreases with an increase in concentration of Er^3+^. Therefore, it can be concluded that the lattice dimension contracts with doping of erbium ions in the calcium substitutional sites.

### Scanning electron microscopy and EDX

**Fig. 2:**
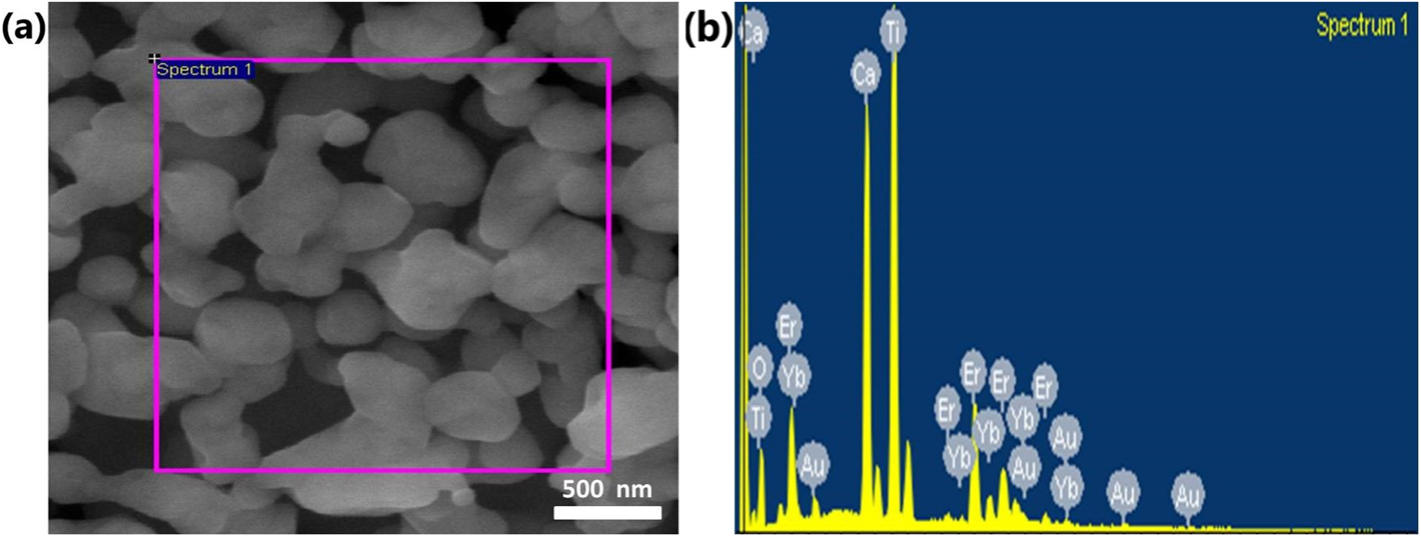
(a) SEM micrographs of the optimized sample CaTiO_3_: 1 at% Er^3+^, 1 at% Yb^3+^, (b) corresponding EDX.

Fig. 2(a) illustrates the surface morphology of the prepared materials using SEM imaging, which was taken using a TESCAN MIRA. The nanomaterials are spherical when seen from the top and agglomeration is seen in the absence of a surfactant. Fig. 2(b) represents the EDX spectra which confirm the presence of Ti (13.86 atomic%), Ca (5.10 atomic%), O (77.68 atomic%), Er (0.60 atomic%) and Yb (2.77 atomic%). Some traces of Au can be seen, due to the presence of gold in the background. All elements were analyzed under normalized conditions and the data taken after four iterations.

### Transmission electron microscopy

**Fig. 3:**
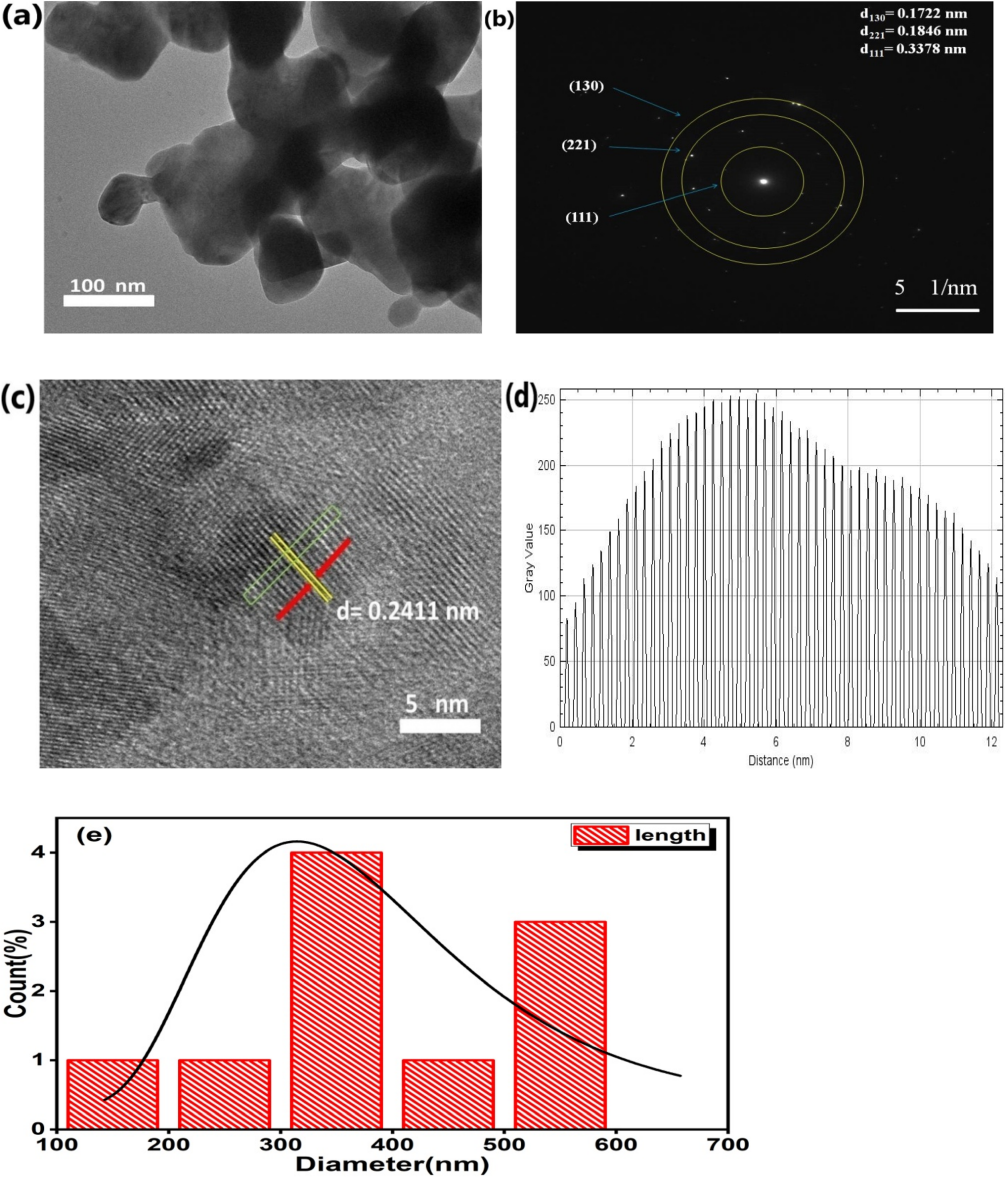
(a) Transmission electron microscopy image of CaTiO_3_: 1 at% Er^3+^, 1 at% Yb^3+^, (b) corresponding SADP, (c) corresponding HRTEM, (d) fringes of lattice spacing and (e) size distribution histogram plot.

The TEM micrograph in Fig. 3(a) confirms the spherical morphology of the particles. It is apparent that the crystallites are agglomerated, which may be due to annealing performed at high temperature (900 °C). The SAED pattern of the Er^3+^-Yb^3+^codoped CaTiO_3_ nanocomposite confirms its polycrystalline nature and it also consists of three different planes, as shown in Fig. 3(b). One of the plane is illustrated with a *d*-spacing of 0.2411, as shown in the HRTEM image. The *d*-spacing values of the resulting diffraction pattern are *d*_111_ = 0.3378 nm, *d*_221_ = 0.1846 nm, and *d*_130_ = 0.1722 nm, and these *d*-spacing values are in good agreement with the XRD studies and are consistent with standard JCPDS file number 76-2400. The presence of crystalline erbium and ytterbium inside the CaTiO_3_ matrix is confirmed by means of the well-defined lattice fringes in Fig. 3(c). FIJI IMAGE-J software was used to analyze the *d*-spacing, which resulted in a *d*_*hkl*_ value of 0.2411 nm. The *d*_*hkl*_ value was estimated using the line profile approach by taking an average of 51 fringes. The histogram plot in Fig. 3(e) shows the Gaussian distribution, which is indicative of a mean particle size of 381.27 nm.

### Upconversion emission spectra

**Fig. 4:**
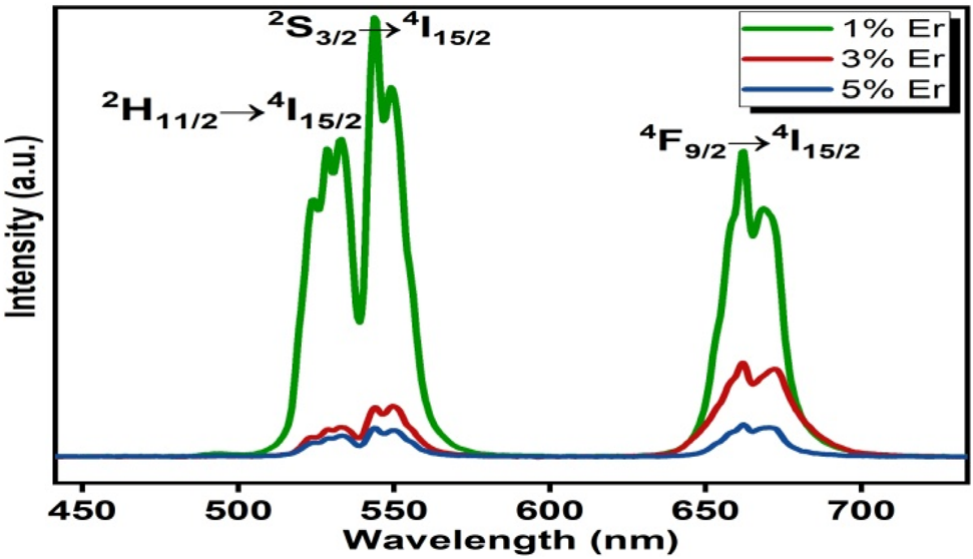
Upconversion spectra of (Ca_0.99−*a*_Yb_0.01_Er_*a*_)TiO_3_ (where *a* = 0.01, 0.03, 0.05).

The upconversion spectra were recorded over the wavelength range 450–750 nm for three different concentrations of Er^3+^ ion. Two intense green UC bands at 523 nm and 544 nm were obtained and assigned to the ^2^H_11/2_ → ^4^I_15/2_ and ^4^S_3/2_ → ^4^I_15/2_ transitions, respectively. An intense red band corresponding to the ^4^F_9/2_ → ^4^I_15/2_ transition at 662 nm was also found. From Fig. 4 it is clearly observable that the emission intensity is a maximum for 1 molar% erbium ion concentration. Owing to concentration quenching, the emission intensity is very low for higher concentrations.^42,43^ Our research goes along with the idea put forward by Wang and co-workers^44^ that at lower Er^3+^ concentrations, approximately twice the optical temperature sensitivity is observed compared to a highly doped concentration. So we continued our other studies for the sample giving the maximum signal.

### Power-dependent upconversion spectra

**Fig. 5:**
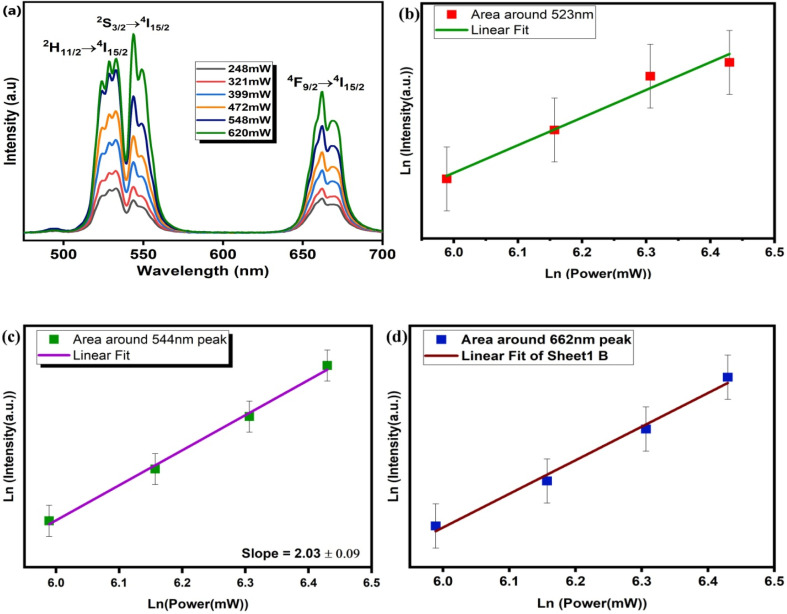
(a) Pump-power-dependent UC spectra for CaTiO_3_: 1 at% Er^3+^, 1 at% Yb^3+^ and (b–d) logarithmic plots for 523, 544, and 662 nm peaks, respectively.

Fig. 5(a) illustrates the pump-power-dependent UC emission spectra. The graph shows a gradual increase in the intensity of UC emission along with an increase in pump power, which establishes the direct dependence of emission intensity on pump power. Here a remarkable change observed in UC emission is that up to a pump power of 548 mW, the highest intensity is observed for 522 nm, but with a further increase in power, the dominant intensity is observed for 544 nm. This might be because up to a power of 548 mW the transition ^2^H_11/2_ → ^4^I_15/2_ is dominant because the population in the ^2^H_11/2_ state is a maximum. However, as the power increases to 620 mW thermal-vibration increases, so that due to non-radiative transition between the ^2^H_11/2_ and ^4^S_3/2_ states and further to the ^4^I_15/2_ ground state, the highest UC emission is observed for 544 nm. The number of photons absorbed can be calculated from the relation below ^45^

3 *I*_uc_ ∝ (*P*_pump_)^*n*^

where the term *I*_uc_ denotes the UC emission intensity, *P*_pump_ denotes the infrared excitation pump-power and *n* stands for the number of photons absorbed.^46^Fig. 5(b)–(d) show the slopes of ln(UC intensity) *vs.* ln(pump power), which confirm the involvement of the two-photon absorption process for the aforementioned upconversion emission bands. The slope of intensity at the 523 nm peak at different pump powers fitted linearly is 1.64 ± 0.19, while for the 544 nm peak the corresponding slope value is 2.03 ± 0.09 and for the peak at 662 nm the corresponding value of slope is 1.67 ± 0.11.

**Fig. 6:**
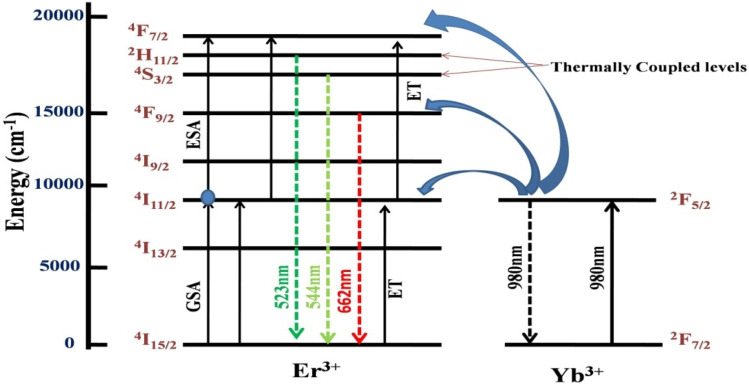
A possible schematic energy level illustration for the characteristic Er^3+^-Yb^3+^ emissions under 980 nm excitation.

Fig. 6 shows a schematic representation of possible absorption and emission pathways for Er^3+^-Yb^3+^ codoped CaTiO_3_. The pump-power-dependent study confirms that a two-photon absorption process took place through successive absorption of two photons mediated through intermediate levels and few non-radiative relaxations. The Er^3+^ ion has an ^4^I_11/2_ energy level which gets populated through the ground state absorption (GSA) method absorbing a 980 nm pump photon and from there the ^4^F_7/2_ level is populated through absorption of another photon of the same energy by the excited state absorption (ESA) process. The photons in the ^4^F_7/2_ state relax non-radiatively to the ^2^H_11/2_ and ^4^S_3/2_ levels and further relax back to the ground state by the emission of visible green photons around 525 nm and 547 nm, respectively. A red emission centered at 662 nm arises because of ^4^F_9/2_ → ^4^I_15/2_ transition in Er^3+^. The Yb^3+^ ions keep enriching the population in all of the emitting levels by the efficient energy transfer (ET) channels shown in the figure. This transfer of energy from Yb^3+^ to Er^3+^ intensifies the emissions correspondingly. Whereas the green color emitting levels, ^2^H_11/2_ and ^4^S_3/2_, have a very close energy separation of Δ*E* = 707.43 cm^−1^, which helps the ^4^S_3/2_ level to keep the ^2^H_11/2_ level thermally populated; this theory was also supported in many earlier studies.^47–52^

### CIE diagram

**Fig. 7:**
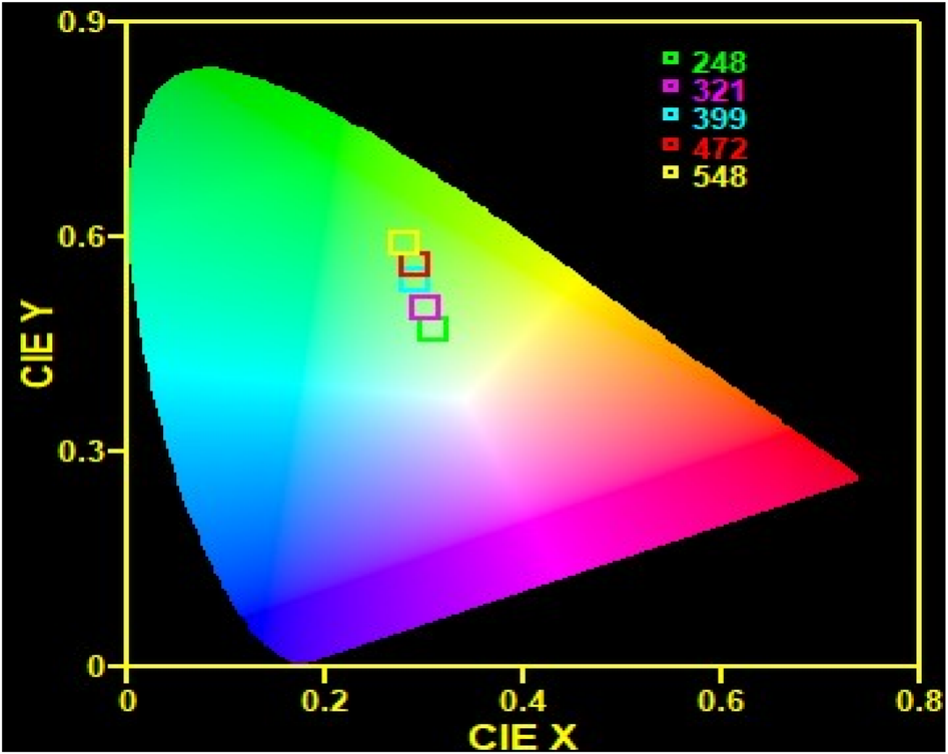
CIE color coordinates for CaTiO_3_: 1 at% Yb^3+^, 1 at% Er^3+^.

Fig. 7 represents a chromaticity diagram of the synthesized nanophosphor under 980 nm excitation. To characterize the color of the visible light emitted from a phosphor, CIE color coordinates were instituted,^53^ which can be calculated separately for different pump powers. As shown in Fig. 7, the color coordinate points fall in the yellowish-green region due to the presence of strong red emission. The obtained color coordinates at different excitation powers are tabulated in Table 1. With increasing pump power, the color coordinates shift towards the green region.

**Table 1:** The color coordinates corresponding to different pump powers at 980 nm excitation

Serial number	Pump power (mW)	CIE coordinates
1	248	(0.31, 0.47)
2	321	(0.30, 0.50)
3	399	(0.29, 0.54)
4	472	(0.29, 0.56)
5	548	(0.28, 0.59)

### Temperature-dependent UC spectra

**Fig. 8:**
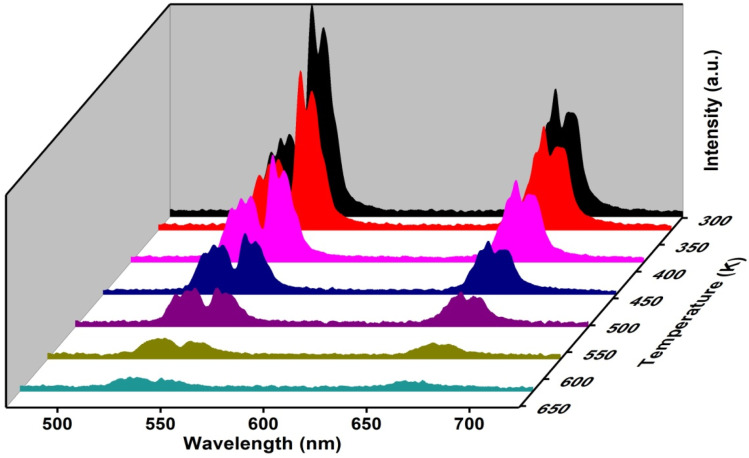
Temperature-dependent UC spectra of (Ca_0.98_Yb_0.01_Er_0.01_)TiO_3_, *λ*_ex_ = 980 nm.

The UC spectra of the as-synthesized samples were observed by varying the temperature at very low pump power to avoid self-heating. The variation in intensity of the green emission bands peaking at 523 nm and 544 nm owing to ^2^H_11/2_ → ^4^I_15/2_ and ^4^S_3/2_ → ^4^I_15/2_ transitions is useful in optical thermometry. In Fig. 8 temperature-dependent emission spectra are shown over the 298–623 K temperature range.

### Fluorescence intensity ratio

**Fig. 9:**
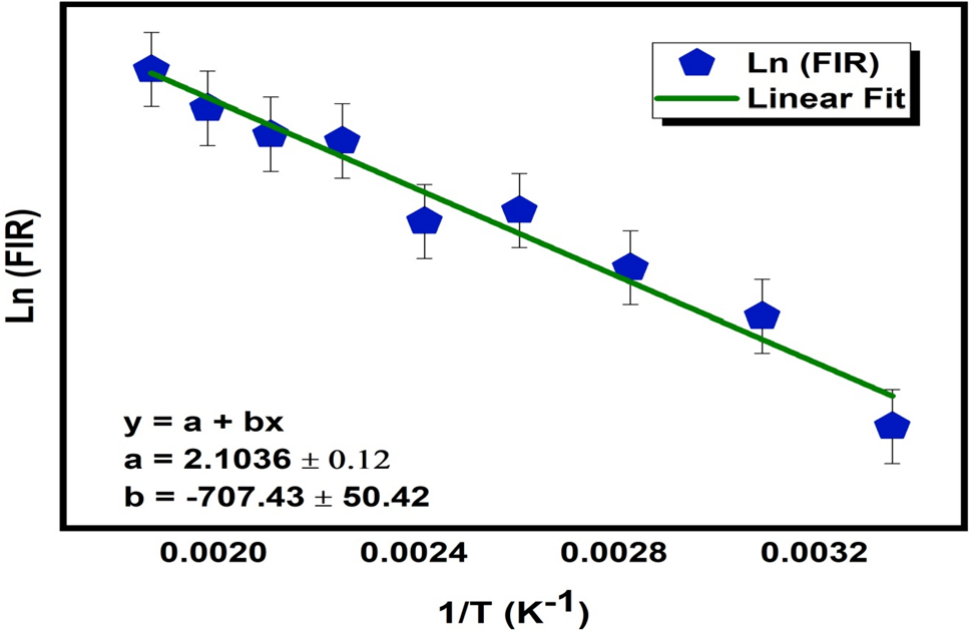
A Plot of variation of ln(FIR) *vs.* inverse temperature for a (Ca_0.98_Yb_0.01_Er_0.01_)TiO_3_ phosphor.

For the calculation of FIR of two thermally coupled levels, ^2^H_11/2_ and ^4^S_3/2_, the Boltzmann distribution expression is used:^54^4
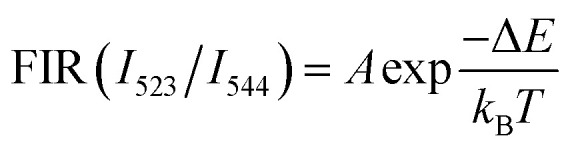
where the terms *I*_523_ and *I*_544_ represent the corresponding intensities, ^2^H_11/2_ → ^4^I_15/2_ and ^4^S_3/2_ → ^4^I_15/2_, respectively. *T* is the absolute temperature, *A* is a pre-exponential constant, Δ*E* is the energy gap between thermally coupled levels (^2^H_11/2_ and ^4^S_3/2_), and *k*_B_ represents the Boltzmann constant. FIR for a (Ca_0.98_Yb_0.01_Er_0.01_)TiO_3_ phosphor has been calculated over the 298–623 K range at constant pump power and is plotted in Fig. 9. A logarithmic plot of FIR *versus* inverse absolute temperature is plotted to estimate the energy gap (Δ*E*) between the two thermally coupled levels, as shown in Fig. 9. The obtained data points of FIR *vs.* inverse *T* were fitted linearly to obtain the energy gap (Δ*E*), which was found to be ∼707.43 cm^−1^, which is almost equal to the experimentally measured energy gap (Δ*E*_m_ ∼700 cm^−1^). For a temperature-sensing application, it becomes very necessary to identify the variation of FIR with varying temperature. The formula below is used to obtain absolute sensitivity (*S*_A_):5
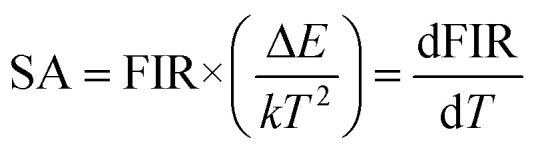


Eqn (5) measures the value of sensor sensitivity. The maximum absolute sensitivity for the present material is found to be 6.71 × 10^−3^ K^−1^ at 383 K which is amongst the good measured sensitivities for luminescent temperature sensors. In Table 2 the sensor sensitivity of the present sample is compared with other reports.

**Table 2:** Maximum temperature sensor sensitivities of different RE^3+^ doped/codoped systems calculated using the FIR algorithm

Rare earth doped materials	Maximum sensitivity (K^−1^)	Temperature range (K)	Excitation wavelength	References
Er/Yb:Na_0.5_Bi_0.5_TiO_3_	3.1 × 10^−3^ K^−1^ at 400 K	163–613 K	980 nm	55
Gd_2_O_3_:Er^3+^-Yb^3+^	3.9 × 10^−3^ K^−1^ at 300 K	300–900 K	976 nm	56
Er:BZT-BCT	4.4 × 10^−3^ K^−1^ at 443 K	200–443 K	980 nm	57
Er^3+^/Yb^3+^ codoped oxyfluoride glass	3.9 × 10^−3^ K^−1^ at 513 K	291–450 K	980 nm	58
CaTi_4_O_9_:Er^3+^, Yb^3+^	4.9 × 10^−3^ K^−1^ at 323 K	303–553 K	980 nm	59
CaMoO_4_:Ho–Yb–Mg	6 × 10^−3^ K^−1^ at 353 K	303–543 K	980 nm	60
Na_0.82_Ca_0.08_Er_0.16_Y_0.853_F_4_:Er^3+^	2.2 × 10^−3^ K^−1^ at 338 K	5–300 K	1.54 μm	61
CaTiO_3_:Er^3+^, Yb^3+^	6.71 × 10^−3^ K^−1^ at 383 K	298–623 K	980 nm	Present work

## Conclusion

Frequency upconversion emission spectra were recorded for an Er^3+^, Yb^3+^ codoped CaTiO_3_ nanophosphor with orthorhombic phase synthesized successfully through a sol–gel technique, and it emerged as an interesting candidate for optical thermometry. The presence of nanoparticles and small clusters was investigated through TEM and HRTEM images. On codoping with Yb^3+^ and increasing the concentration of Er^3+^, the intensity of the emission bands starts decreasing, which confirms the concentration quenching effect. The spectra of UC emission for a temperature-dependent study of the above nanophosphor corresponding to the ^2^H_11/2_ → ^4^I_15/2_ and ^4^S_3/2_ → ^4^I_15/2_ transitions over a wide temperature range of 298–623 K show a maximum sensor sensitivity of 6.71 × 10^−3^ K^−1^ at 383 K. It can be concluded on the basis of the experimental observations that the present phosphors could be excellent candidates for a temperature-sensing probe, in near infrared to green light emitting devices.

## Conflicts of interest

There are no conflicts to declare.

## Supplementary Material
